# Association Between Physical Activity Level, Quality of Life Determinants, Internet Use, and Orthorexia Among Sport Science Students Living in Naples: An Observational Study

**DOI:** 10.3390/healthcare14030369

**Published:** 2026-01-31

**Authors:** Daniela Vitucci, Sara Dei, Rosa Ghirelli, Agnese Turi, Domenico Martone, Andreina Alfieri, Stefania Orrù, Annamaria Mancini, Pasqualina Buono

**Affiliations:** 1 Department of Medical, Human Movement and Wellbeing Sciences, University Parthenope, 80133 Naples, Italy; daniela.vitucci@uniparthenope.it (D.V.); sara.dei@studenti.uniparthenope.it (S.D.); ghirelli@ceinge.unina.it (R.G.); andreina.alfieri@uniparthenope.it (A.A.); stefania.orru@uniparthenope.it (S.O.); pasqualina.buono@uniparthenope.it (P.B.); 2 CEINGE-Biotecnologie Avanzate “Franco Salvatore”, 80145 Naples, Italy; turi@ceinge.unina.it; 3 Department of Neurosciences, Biomedicine and Movement Sciences, University of Verona, 37129 Verona, Italy; 4 Department of Economics, Law, Cybersecurity and Sport Sciences, University Parthenope, 80133 Naples, Italy; domenico.martone@uniparthenope.it

**Keywords:** sport science university students, physical activity, quality of life, orthorexia nervosa, internet use

## Abstract

Background: In recent years, growing attention has been paid to the lifestyle factors that influence young adults’ well-being. University students represent young adults at risk of Sedentary Behavior (SB) and mental distress. Sport Science students represent a health-conscious population, less prone to mental distress. This study aims to investigate the associations between physical activity (PA) levels, different determinants of quality of life (QoL), orthorexia nervosa (ON) symptoms, and internet use among Sport Science students living in Naples. Methods: An online survey comprising General Data (GD) and eight validated questionnaires was used to assess PA levels, mood, sleep quality, eating habits, and digital behavior in a population of university students enrolled in Sport Science courses at Parthenope University, Naples. The statistical analyses included descriptive statistics, Student’s t-test, a Mann–Whitney U Test, frequencies, *chi-square* tests, and a Spearman’s rank correlation. All the analyses were performed using JASP and Jamovi software. Results: We surveyed 775 students (472 M; 303 F; 22.85 ± 3.85 y; BMI 23.74 ± 3.63 kg/m^2^). Regarding the MET-min/week, 65% of participants reported being highly active, 28% moderately active, and 7% inactive. Poor sleep quality was reported by 20% of those surveyed. Additionally, 84% of participants declared average internet use, which positively correlated with their emotional profile and sleep quality. High PA levels were directly associated with the presence of ON symptoms in 27% of the participants, most of whom exercised in gyms. Conclusions: To our knowledge, this is the first study conducted on a study population of Sport Science University students addressing the complex and interconnected relationships between PA levels, QoL, ON symptoms, and internet use.

## 1. Introduction

Robust evidence supports the beneficial effects of PA on health across several domains, including all-cause mortality; cancer; and cardiovascular, musculoskeletal, metabolic, and neurocognitive health [1,2].

On the other hand, SB and physical inactivity are associated with an increased risk of several chronic diseases, including heart disease, chronic obstructive pulmonary disease, diabetes, hypertension, osteoporosis, depression, and several types of cancer [3,4]. SB is defined as any waking behavior characterized by an energy expenditure of 1.5 metabolic equivalents (METs) or less, performed while in a sitting or reclining posture (e.g., sitting, watching television, or computer use) [5,6].

The 2020 WHO guidelines on PA and SB for adults (18–64 years) state that all adults should engage in regular PA, as some activity is better than none [1]. These guidelines for adults include strong recommendations regarding weekly volumes of aerobic PA and muscle-strengthening activity. Many of the benefits of PA are seen with average weekly volumes of 150–300 min of moderate intensity or 75–150 min of vigorous intensity, or an equivalent combination of moderate–vigorous PA (MVPA) [7,8].

Given that the most recent global estimates show that one in four adults (27.5%) [6] and more than three-quarters (81%) of adolescents [9] do not meet the recommendations for aerobic exercise, and that in Italy, less than one in two adults (aged 18–69) achieves sufficient levels of PA [10], painting a worse picture in Italy than compared to global trends, it is crucial to raise awareness and increase action regarding health and other key areas (e.g., regular PA).

Alongside the concept of PA, quality of life (QoL) has been established as a significant focus of research and practice in the fields of health and medicine [9]. QoL is a complex and multifaceted concept that has been used to study several aspects of peoples’ lives, including physical and psychological well-being, financial independence, social relationships, personal beliefs, and living situation [10]. The WHO defines QoL as “individuals’ perception of their position in life in the context of the culture and value systems in which they live and in relation to their goals, expectations, standards and concerns” [7].

Early adulthood is one of the most crucial phases of human development [8]. For this population, the transition from secondary to higher education can be especially demanding for many reasons, including the pressure to thrive academically, competition between peers, changes in workload and support networks and, at times, changes in living conditions and being away from family for extended periods [11].

Emerging evidence suggests that college students’ lifestyles are closely interrelated, with implications for both their physical and mental health, including anxiety and depression [12,13]. Low levels of PA and prolonged SB have been associated with poorer QoL, including increased stress, fatigue, and decreased social skills [14]. At the same time, excessive internet use has been linked to increased time spent sitting, and an increased risk of ON [15,16]. ON, characterized by an obsessive focus on ‘healthy’ eating, can be exacerbated by exposure to online health videos and fitness content, particularly in populations already vulnerable to lifestyle pressures [17]. Therefore, it is appropriate to investigate the relationships between PA, QoL, internet use, and ON in university students, as these factors may influence their overall well-being and the risk of maladaptive behaviors [18].

Given the content of their academic degrees, Sport Science students should be aware of the benefits of regular PA and, conversely, the risks associated with SB. Further, their well-being should be evaluated through the lens of a 24 h movement behavior framework, which emphasizes the integrated relationship between PA, sedentary time, and sleep [19,20,21].

However, until now no study has addressed the associations between PA levels, QoL determinants, internet use, and ON symptoms in university students engaged in human movement and Sport Sciences living in southern Italy. Due to the fact that QoL encompasses interconnected physical, psychological, and behavioral dimensions, this research adopts a multidimensional approach to examine how engagement in PA may influence well-being in young adults. We constructed an online survey containing GD and eight sections composed of validated questionnaires: Global Physical Activity Questionnaire (GPAQ), Short Form 12 Health Survey (SF-12), Profile of Mood States (POMS), Pittsburgh Sleep Quality Index (PSQI), Oswestry Disability Index (ODI), Mediterranean Diet (MD) Adherence, Internet Addiction Test (IAT), and Italian-Düsseldorf Orthorexia Scale (I-DOS) to assess the physical health, moods, sleep quality, eating habits, and digital behavior of university students engaged in Sport Science courses at Parthenope University, Naples.

By integrating these domains, the study seeks to provide a comprehensive understanding of the relationship between PA levels and overall well-being within a population of emerging adults engaged in sports-related academic training.

## 2. Materials and Methods

The authors have read the STROBE Statement checklist of items, and the manuscript was prepared and revised accordingly.

### 2.1. Questionnaire Survey

From 8 January to 25 February 2025, we administrated the questionnaire survey to students enrolled in a Bachelor’s or Master’s Degree in Sports Science for Prevention and Health/Sport and Management courses at Parthenope University (Naples). The survey was anonymous and voluntary; the completion and submission of the questionnaire implied informed consent for the processing of data (See 2.12. Ethics and Informed Consent). To facilitate the participants’ comprehension and understanding, the survey was written and administered in Italian. The Google Forms setting allowed only one response per user. The participants were recruited during class sessions, where they received detailed information regarding the study. The researchers provided a digital link to the survey and guaranteed complete anonymity for all submitted data. The questionnaire was completed independently on the participants’ own devices (e.g., smartphones or laptops) at their convenience, without the constraints of a classroom setting. The researchers emphasized the importance of having adequate time available to complete the 119-items survey. Of the 902 invited students, we received 779 answers that represent an 86.4% response rate; 4 answers were excluded due to inconsistent and incongruent data between the GD and GPAQ parts. So, we analyzed 775 surveys (472 males and 303 females). Among the participants, the “Other” category (referred to in the “Academic Degree engagement” section of Table 1) included 7 participants (4 males, 3 females). They recently graduated with a Master’s Degree in Sport Science and were unemployed at the time of data collection.

### 2.2. General Data (GD)

We used the following online Google Forms composed of a total of 119 items, subdivided into nine sections: a brief introduction assuring the anonymity and the non-collection of sensible personal data (e.g., names, home addresses, email addresses, and phone numbers); (1) GD; (2) GPAQ [22]; (3) SF-12 [23]; (4) POMS [24]; (5) PSQI [25]; (6) ODI [26]; (7) Adherence to MD [27]; (8) IAT [28]; and (9) I-DOS [29]. All questionnaires were administered in their validated Italian versions (See Supplementary Materials for complete survey module). The GD section comprises twelve questions aiming to frame the responders from their demographic, self-reported anthropometric, and sport practice aspects. In order, the questions ask about their study degree program; home city; age (in years); gender; height (in m); weight (in kg); present practiced sport; sport frequency per week; single session volume (in min); sessions a day; training intensity (detailed below); and sport experience (in years).

The training intensity is divided into five answer choices: low, moderate, vigorous, high intensity, or do not train. Each training intensity has a description providing the related METs equivalent range and a practical explanation. MET is defined as the amount of energy consumed while sitting at rest and it is equivalent to a caloric consumption of 1 kcal/kg/hour [30]. Low intensity ranges from 1.6 to 3 METs and refers to aerobic activity that does not cause significant changes in respiratory frequency. Moderate intensity ranges from 3 to 6 METs and refers to aerobic activity that generally can be protracted while speaking. Vigorous intensity ranges from 6 to 9 METs and refers to aerobic activity that generally cannot be protracted while speaking. High intensity is higher than 9 METs and refers to an activity that cannot be performed for more than ten minutes [31,32].

### 2.3. Global Physical Activity Questionnaire (GPAQ)

The GPAQ is a modified version of Internet Physical Activity Questionnaire (IPAQ), and it was developed by the WHO in 2002 in response to the great interest in the role of PA in health. The original version of the GPAQ contained nineteen questions with a shorter version developed later, which eliminated three redundant questions [12]. The GPAQ has a total of sixteen questions (P1-P16) that assess SB and three domains in which PA is performed: work, transport, and leisure-time activities. It covers several components of PA, such as intensity, duration, and frequency. Intensity of PA is expressed in terms of MET.

The study participants were asked if they engaged in vigorous and moderate work or leisure-time activities continuously for at least 10 min. They provided answers about the number of days they engaged in each activity in a typical week, and the time spent on each activity on a typical day. The scoring system attributes 8 MET-min to vigorous-intensity activities; 4 MET-min are attributed to moderate-intensity activities. SB is assessed through the question “How much time do you usually spend sitting or reclining on a typical day?” [33]. The total score is expressed in MET-min/week and derives from the following equation.

(1) $$\mathrm{MET}\frac{\min}{\mathrm{week}}=\left(P2\times P3\times 8\right)+\left(P5\times P6\times 4\right)+\left(P8\times P9\times 4\right)+\left(P11\times P12\times 8\right)+\left(P14\times P15\times 4\right)$$

According to the WHO GPAQ Analysis Guide [34] and latest reference about the interpretation of the scoring system for healthy people [35], individuals are divided into three classes: “Low PA” (inactive) if they achieve <600 MET-min/week, “Moderate PA” (active) if they achieve between 600 and 2999 MET-min/week, and “High PA” (highly active) if they achieve ≥3000 MET-min/week.

### 2.4. Short Form 12 Health Survey (SF-12)

The SF-12 is one of the most widely used instruments for assessing self-reported health-related quality of life (HRQoL). It was originally developed from the Medical Outcomes Study as a 36-item Short-Form Health Survey (SF-36). The SF-12 consists of twelve questions that measure eight health domains to assess physical and mental health. The physical health-related domains include General Health, Physical Functioning, Role—Physical, and Body Pain. The mental health-related domains include Vitality, Social Functioning, Role— Emotional, and Mental Health [36]. The eight domain scores are summarized into two component summary scores: Physical Component Summary (PCS) score and Mental Component Summary (MCS) score.

The norm-based PCS and MCS scores for the general population are 50 ± 10. Higher scores indicate better health status [37]. According to the scoring system, individuals are divided into two classes: a score < 20 means “frequent psychological unease” and a score ≥ 20 means “psychological health” [38].

### 2.5. Profile of Mood States 11-Items Short Version (POMS)

The POMS is a valid and reliable instrument that has been widely used in clinical and psychological research. There are full length and shorter versions of the POMS suitable for different research environments. The POMS Short Form has thirty-seven items divided among six subscales: Anger, Confusion, Depression, Fatigue, Tension, and Vigor. The responders indicate how much they have felt like the word (adjective) in the last two weeks on a Likert scale (0 = not at all; 1 = a little; 2 = moderately; 3 = quite a bit; 4 = extremely) [39]. In our study we used the brief version of POMS 11-items proposed by Cella and colleagues, which shows good internal consistency with the Total Mood Disturbance (TMD) score, a reliable index for QoL [40]. The following equation gives the POMS TMD score.

(2) $$\mathrm{TMD}=\left(\mathrm{sum}\;\mathrm{of}\;\mathrm{negative}\;\mathrm{adjectives}\right)-\left(\mathrm{sum}\;\mathrm{of}\;\mathrm{positive}\;\mathrm{adjectives}\right)$$

Following the scoring system, score range from 0 to 100 points and individuals are divided into three classes according to their TMD score. A score < 40 means “lack of emotional sensitivity”, a score between 40 and 60 means “normal emotional area”, and a score > 60 means “potentially problematic emotional area” [40].

### 2.6. Pittsburgh Sleep Quality Index (PSQI)

The PSQI is a widely used self-report questionnaire that assesses sleep quality over a one-month interval. The PSQI is commonly used in both clinical and research settings to evaluate various aspects of sleep, including both subjective experiences and objective parameters. It allows researchers and healthcare providers alike to obtain a comprehensive understanding of an individual’s sleep patterns and disturbances (e.g., trouble getting to sleep, going to the toilet frequently). The PSQI’s strong validity is supported by its consistent performance in identifying individuals with sleep disturbances using a post hoc cutoff score. A comparative analysis with polysomnography further substantiated the PSQI’s validity, showing alignment with objective measures of sleep across various subject groups [41].

The PSQI includes nineteen questions, categorized into seven groups that collectively make up a global sleep quality score. This aggregate score is derived from questions relating to “sleep quality, sleep latency, sleep duration, habitual sleep efficiency, sleep disturbance, use of sleeping medication and daytime dysfunction.” The PSQI items use different response categories that include recording the usual bedtime, usual wake time, the number of hours slept, and the number of minutes to fall asleep [42].

Each constituent question produces a score on a scale from 0 to 3, with 3 indicating the greatest dysfunction. The total score is made up of scores from each of the seven subgroups of questions, giving a cumulated score between 0 and 21, with lower scores indicating better sleep quality and higher scores indicating poorer sleep quality [41]. If the PSQI score is less than 5, it is inferred that the subject has a good quality of sleep, and vice versa for higher scores (poor sleep quality). These scoring criteria were re-based on multiple studies using a range of population samples, including college responders, and are believed to be generalizable to the current sample studied [43].

### 2.7. Oswestry Disability Index (ODI)

The ODI has become one of the principal condition-specific measures used in the management of spinal disorders. The development of the ODI was initiated by John O’Brien in 1976 [44]. The questionnaire is formed of ten questions (items) asking about the possible limitations/disabilities due to chronic low back pain. The items ask about pain intensity, limitations to personal care, limitations to lifting heavy things, limitations to walking, limitations to sitting, limitations to standing, limitations to sleeping, limitations to sexual life, limitations to social life, and limitations to travel. Each item is scored on a Likert scale of 0–5 points [45].

The total limitation score is formed by the sum of the ten items. Scores range from 0 to 50 points, and the population is classified into four classes. If a subject’s score is 0–4, they have “no disability” due to back pain; a score of 5–14 means “mild disability”; a score of 15–24 means “moderate disability”; a score of 25–34 means “severe disability”; and a score of 35–50 means “complete disability” [44].

### 2.8. Adherence to Mediterranean Diet (MD)

The MD has the advantage of a very lengthy history. It has been used for centuries, even in resource-poor areas, which argues in favor of its potential adoption across the world. Moreover, in more recent times evidence has accumulated for the health benefits of the MD [46]. Adherence to the MD is defined thorough scores that estimate the conformity of the dietary pattern of a population with the traditional Mediterranean dietary pattern [47]. The MD pattern includes consumption of fruit (>2 portions/day), vegetables (>2.5 portions/day), legumes (2 portions/week), cereals (>2 portions/day), fish (>2.5 portions/week), meet and cured meats (<1 portion/day), milk and dairy products (<1 portion/day), alcohol (1–2 Alcoholic Unit/day), and olive oil (frequently).

The MD scoring system range is 0–18 points. Each of the nine items has a maximum score of 2 points, assigned to the appropriate food consumption, as previously described. Otherwise, for each item, the subject is given 1 or 0 points according to the distance between the recommended consumption. A total adherence to the MD score is formed by the sum of each item score and the population is divided into three groups as follows: a score of 0–8 indicates “low adherence”, a score of 9–10 indicates “medium adherence”, and a score of 11–18 indicates “high adherence” [47].

### 2.9. Internet Addiction Test (IAT)

The IAT measures the use of electronic devices with online access (e.g., mobile phones, computers). Young described internet addiction as “an impulse-control disorder which does not involve an intoxicant” [48]. Later, Young extended the previous version of IAT. The new scale exhibits the following characteristics:

- The IAT comprises 20 items rated on a five-point Likert scale (from 1—not at all to 5—always);
- As with the first diagnostic questionnaire, this measurement is derived from the DSM-IV (*Diagnostic and Statistical Manual of Mental Disorders IV*) criteria for pathological gambling and alcoholism, and it measures the extent of an individual’s problems with daily routines, social life, productivity, sleeping patterns, and feeling due to internet use [49].

The questionnaire examines the degree of preoccupation, compulsive use, behavioral problems, emotional changes, and the impact on life related to internet use. The IAT score ranges from 0 to 100 points with the following cut-offs. A score of 0–39 is for an “average online user”, a score of 40–70 is for a “problematic online user”, and a score of 71–100 is for an “addictive online user” [50].

### 2.10. Italian-Düsseldorf Orthorexia Scale (I-DOS)

In the 1990′s, Bratman described for the first time orthorexia nervosa (ON) as an extreme level of preoccupation around healthy eating, accompanied by restrictive eating behaviors. It can be considered a disorder to the extent that the pursuit of healthy food negatively impacts upon other areas of life, such as work and relationships, and it is associated with significant changes in lifestyle [51]. The Düsseldorf Orthorexia Scale has been validated in different languages, and it has shown good reliability, criterion validity, and factor structure [52].

The I-DOS scale consists of 10 items assessing ON behaviors and attitudes, using a 0–1 point scale. The score range is 0–10. If a score is between 0 and 3, symptoms of ON are absent; if score is between 4 and 10, symptoms are present [51].

### 2.11. Statistical Analyses

The statistical analyses were performed using JASP (Version 0.95.3.0; JASP Team, Amsterdam, Netherlands) and Jamovi (Version 2.3.26.0; Jamovi Project, Sydney, Australia) software. Descriptive statistics were produced for the preliminary analysis. A Shapiro–Wilk statistical test was used to check the normality of the distributions. The variables that had a non-parametric distribution were expressed as medians and the interquartile range, while those that had a normal distribution were expressed as the mean and standard deviation. A Student’s *t*-test was used to compare the anthropometric variables between males and females. A Mann–Whitney U test was applied for the training-related variables. For all the analyses, statistical significance was set at *p* < 0.05. The gender influence on the questionnaire scores was assessed by a *chi-square* (χ^2^) test, with Cramer’s V calculated to determine the effect size. A power analysis was conducted using the G*Power program (version 3.1.9.7 for Windows) to test the research hypothesis at a 0.05 significance level (type I error rate), assuming a minimally interesting effect size (δ = 0.20) and a minimum desired power of 0.80. A Spearman’s rank correlation was applied to assess the association between the variables of interest. The correlation between two variables is denoted by Spearman’s rho and quantified by a number, which varies between −1 and +1: 0.1 ≤ rho ≤ 0.3 means a weak association; 0.4 ≤ rho ≤ 0.6 means a moderate association; and 0.7 ≤ rho ≤ 0.9 means a strong association [53]. Zero means there is no correlation; ±1 means a complete or perfect positive or negative correlation. The direction of Spearman’s rho in this study should be interpreted according to the rank assignment. Consequently, the sign of the correlation reflects this numerical coding and has been interpreted based on the substantive direction of the observed phenomena [54].

### 2.12. Ethics and Informed Consent

The data collection was conducted anonymously, ensuring participant confidentiality and preventing any identification [55,56]. Anonymity was ensured by excluding any personal data in the questionnaire.

Further, the study was approved by the Dean of the Department; all the data were analyzed in aggregated form, according to Italian and European laws on personal data protection [57,58,59]. We also provided the purpose of the study with the presentation of the questionnaire; by clicking the link to complete the survey, the volunteers confirmed their understanding of the study procedures and objectives and provided informed consent for both their participation and data collection and processing.

Participation was exclusively voluntary, and the participants were free to withdraw at any time prior to submission by exiting the questionnaire, ensuring that no partial responses were recorded.

Google Forms was used only for questionnaire distribution and data collection. All the data were subsequently downloaded and securely stored on a password-protected professional computer.

## 3. Results

The participants’ characteristics are summarized in Table 1. Of the 902 invited students, we received 779 answers that represent an 86.4% response rate; 4 were excluded due to inconsistent and incongruent answers to the GD and GPAQ parts. The participants (N = 775) were young adults, 472 males and 303 females, matched for age (22.88 ± 4.01 M; 22.81 ± 3.59 F). Both the males and females were of normal weight according to a BMI analysis (24.68 ± 3.62 M; 22.28 ± 3.15 F). As shown in Table 1, significant sex-related differences were observed for weight, height, and BMI. Most of participants (74.07%) lived in metropolitan areas of Naples and its surroundings; only a limited number (3.10%) were off-site, so they were not natives of Campania region. Regarding the educational and professional status of the participants, 70.84% of students were enrolled in a Bachelor’s Degree in Sport Science, and 28.26% in a Master’s Degree in Sport Science for Health and Prevention/ Sport and Management. The “Other” category included seven participants (four males, three females) who were recent graduates of the Master’s Degree in Sport Science and unemployed at the time of data collection.

More than half of the students regularly engaged in gym activities (52.13%); moreover, 18.58% of participants practiced a ball team sport (e.g., football, basketball, or volleyball). Only 10.06% of students, equally distributed between males and females, were not regularly engaged in any sport (Table 2). The trend for sports practice was to train three times a week (IQR 3–4) for 90 (IQR 60–120) minutes, with a training volume per week of 360 (IQR 240–480), and an experience in the sport of 4 years (IQR 2–10). Significant sex-related differences were observed for training volume and experience, as detailed in Table 2.

In order to test the influence of gender on the questionnaire scores, a *chi-square* (χ^2^) test was used. No gender influence (*p* > 0.05) was found in any of the questionnaire scores completed by the participants (Table 3).

**Figure 1:**
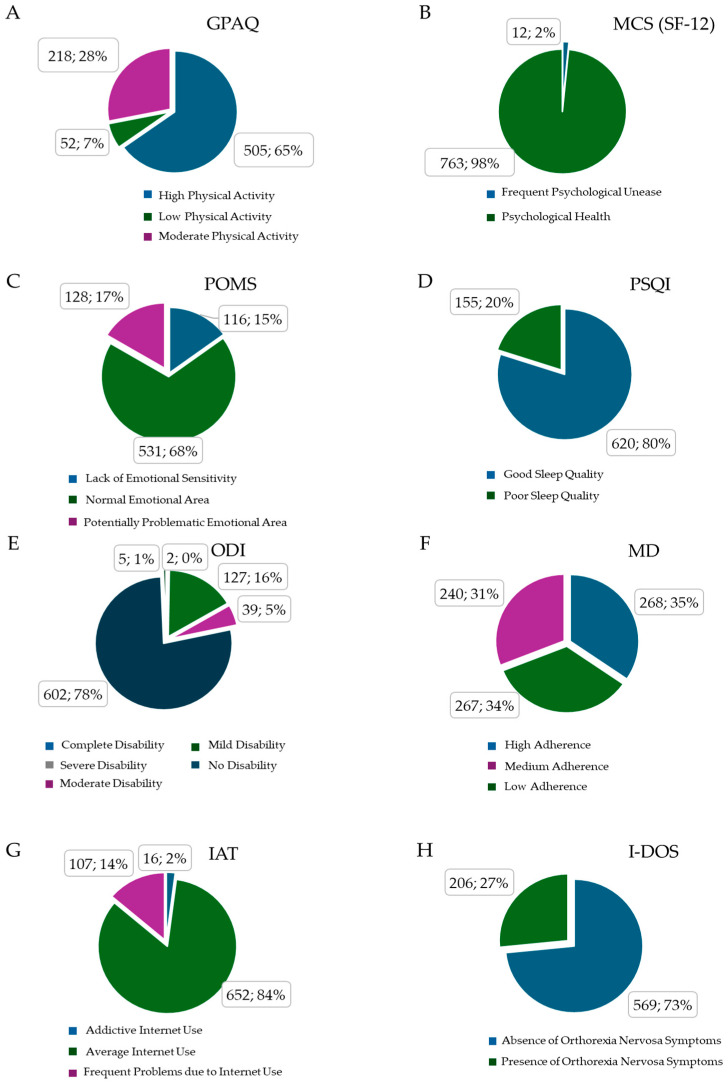
Relative distribution of participants across different questionnaires’ categories. (**A**) GPAQ (Global Physical Activity Questionnaire); (**B**) MCS (Mental Component Summary of Short Form 12); (**C**) POMS (Profile of Mood States); (**D**) PSQI (Pittsburgh Sleep Quality Index); (**E**) ODI (Oswestry Disability Index); (**F**) MD (Mediterranean Diet Adherence); (**G**) IAT (Internet Addiction Test); (**H**) I-DOS (Italian Düsseldorf Orthorexia Scale).

The relative distribution of participants across the different classes for each questionnaire is reported using pie charts (Figure 1A–H). In brief, 65% of students reached a high amount of weekly PA, 28% reached moderate PA, and 7% of participants reached low PA. We opted to consider the PCS of SF-12 as a constant parameter since 100% of participants reported themselves as physically healthy. The MCS of SF-12 revealed that only 2% of this sample had frequent psychological unease. The POMS analysis showed that 68% of participants fell into a normal emotional range, 17% had potentially problematic emotions, and 15% lacked emotional sensitivity. The PSQI score evidenced that 20% of the study population slept badly; on the contrary, 80% reported no trouble with their quality of sleep. The ODI percentages underline that 78% of the participants did not suffer from back pain, 16% reported mild, and 5% reported moderate disability. The MD analysis showed that 35% of the participants strictly followed MD principles, 31% had a medium adherence, and 34% did not follow a MD strictly. The IAT results evidenced that 14% of participants had frequent problems due to internet use; only 2% showed a profile of addiction to online devices. According to the I-DOS results, a substantial 27% of participants met the criteria for ON.

Using a Spearman’s correlation analysis (Table 4), we obtained seven statistically significant correlations (*p* < 0.001) among the tests. The GPAQ negatively correlated with the I-DOS (−0.123); the MCS positively correlated with the POMS (0.221) and PSQI (0.146); the IAT positively correlated with both the POMS (0.170) and PSQI (0.180), and the I-DOS positively correlated with both the PSQI (0.193) and IAT (0.179).

**Figure 2:**
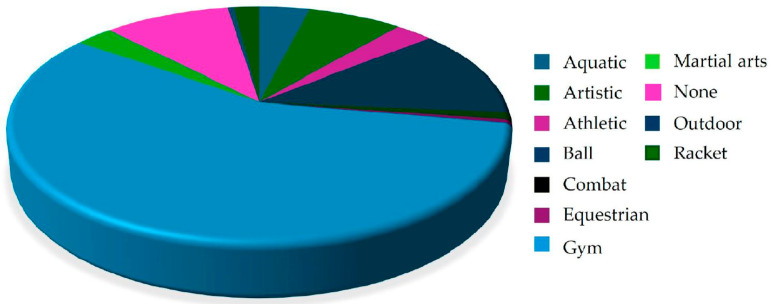
Sports practiced by participants exhibiting symptoms of ON.

Regarding the prevalence of ON symptoms, interestingly, 206 students (27% of the total sample) met the criteria for this condition (Figure 1H). An analysis of their sports activities revealed that 57.07% of these students regularly trained in a gym, while 12.20% were engaged in a team ball sport (Figure 2).

## 4. Discussion

In this study, we sought to determine whether different levels of PA were associated with the different components of QoL, internet use, and ON symptoms among Sports Science students enrolled at Parthenope University (Naples, Italy). A total of 779 answers were received from 902 invited students, yielding a response rate of 86.4%. After data screening, by analyzing 775 participants through a comprehensive set of validated questionnaires (GPAQ, SF-12, POMS, PSQI, ODI, MD, IAT, and I-DOS), we aimed to capture the multifaceted nature of well-being in this population. Our approach allowed us to gain insights into the interaction among lifestyle factors in a group of young adults belonging to an educational environment focused on sport and a healthy lifestyle.

Regular PA and sport engagement are associated with a healthier profile of young populations, such as students [60,61,62]. Although Sports Science students are expected, by virtue of their academic training, to maintain an active lifestyle, recent findings have revealed that SB and compromised psychological well-being are prevalent even in this population [10].

Espada and colleagues showed that athletes are most inclined to eating disorders, as they focus heavily on their weight and diet with the aim of enhancing their performance and physical appearance [63].

In this study, we found that male and female participants met the WHO recommendations, although the males showed a higher volume of weekly training [1].

The students exhibiting ON symptoms (I-DOS questionnaire), characterized by strict attention to the quantity and quality of food consumed, showed a direct association with higher levels of weekly PA (GPAQ questionnaire; notwithstanding the negative rho value due to rank coding). Conversely, ON symptoms were negatively correlated with both good sleep quality and conscious internet use, suggesting that higher orthorexic tendencies are linked to poorer sleep and less regulated internet behavior. Moreover, psychological well-being was positively associated with both a positive emotional profile and good sleep quality. On the other hand, conscious internet use was positively correlated with both a good emotional profile and sleep quality.

Several studies have analyzed the correlation between ON symptoms and PA [64,65]. In particular, Dimitrova [66] and colleagues reported symptoms of eating disorders among university students engaged in higher levels of PA. Similarly, other authors have reported significant positive correlations between ON symptoms and PA volume, measured as the number of weekly exercise sessions between fitness center members and sports participation among university students [67,68]. The 27% ON prevalence rate in our study aligns with previous results reporting an ON prevalence of near 42% among the university students of Parma (Northen Italy) [69], and of near 30% among university students in central Italy (University La Sapienza, Rome), with no significant sex differences [70]. These discrepancies may be attributed to the use of different methods.

To our knowledge, no previous studies have investigated the relationship between PA levels and the different determinants of QoL, MD adherence, internet use, and presence of ON symptoms in a sample of Sport Sciences university students.

We found that approximately 27% of surveyed students exhibited ON symptoms; of these, 57.07% reported training in a gym. This finding highlights the common tendency among young adults who regularly attend a gym to develop excessive attention focus on eating habits. A closer analysis of the data collected shows that the prevalence of ON symptoms was highest among those who exercised four times a week; however, we do not have data regarding the specific type of exercises performed. A perfectionist mindset can lead individuals to use dietary control as a stress-management strategy, a tendency further exacerbated by gym culture’s emphasis on a ‘perfect’ physique [71]. We cannot exclude other factors, such as academic pressure or lifestyle habits, which may influence this prevalence. As suggested by Wachten et al. [72], the context of PA often ‘normalizes’ restrictive eating, making it increasingly difficult to distinguish a healthy commitment from orthorexia tendencies. Interestingly, in our study, adherence to a MD did not seem to influence the QoL determinants.

A recent study found positive relationships between PA and self-rated QoL and inverse associations between PA and perceived stress [16]. The positive link between mental state, mood, and sleep highlights the interconnection between psychological well-being and QoL [73].

Digital addiction often leads individuals to spend long periods indoors, resulting in continuous exposure to blue light emitted by devices both during the day and at night [74]. Extensive research has demonstrated a correlation between average sleep quality, social media use, and mental health in young people [75,76]. In particular, some evidence indicates that excessive engagement with social media platforms is linked to deteriorating sleep quality and increased risk of negative mental health outcomes in young populations [77]. Furthermore, a cross-sectional study conducted across seven countries concluded that internet addiction is a significant predictor of poor sleep quality [74]. Currently, growing evidence supports the view that movement behavior, including PA levels, sedentary time, and sleep duration, are interconnected throughout a 24 h cycle and should therefore be assessed collectively [78]. From this perspective, the associations found in our study between ON symptoms, PA levels, sleep quality, and internet use can be interpreted through a 24 h movement behavior framework [19]. This model indicates that the three categories of movement (PA, SB, and sleep quality) are mutually exclusive and exhaustive components of a 24 h period. Consequently, a change in one of the three categories inevitably affects the others [20]. For instance, the time spent on digital monitoring (problematic internet use) or excessive exercising may directly compromise sleep hygiene, explaining the direct correlation observed between the presence of ON symptoms and poor sleep quality or addictive online use.

## 5. Conclusions

To our knowledge, this is the first study conducted on a population of Sport Sciences university students living in southern Italy to address the complex and interconnected associations among PA levels, QoL determinants, and ON symptoms. Our findings demonstrate an association between high PA levels and the presence of ON symptoms, suggesting that while being more active is generally beneficial, it can sometimes coincide with an excessive focus on controlled food intake. Furthermore, the positive links between mood disorders (POMS), sleep quality (PSQI), and internet use (IAT) reveal how emotional and digital behaviors are closely intertwined, collectively shaping students’ overall well-being. Although these associations are modest, they indicate a coordinated interaction between physical, emotional, and behavioral dimensions.

### Study Limitations

Our study has some limitations. First, the cross-sectional design limits causal inference and the presence of uncontrolled confounding factors, such as academic stress and socioeconomic status, may have influenced the results. Second, the self-reported nature of PA levels via the GPAQ questionnaire is susceptible to recall and social expectation bias. Similarly, the self-reported data regarding eating habits and ON symptoms were not clinically validated, which should be considered when interpreting the results. Furthermore, the research was limited to students attending a single university; it might be appropriate to enlarge the observation to Sport Sciences students belonging to other geographic areas and integrate objective measurement tools (such as accelerometers) in future studies to more accurately assess PA and nutrition levels.

## Figures and Tables

**Table 1:** Baseline characteristics of participants: anthropometric measures, geographic origin, and academic engagement.

	Total (N = 775)	Males (N = 472)	Females (N = 303)
Anthropometry			
Age (years)	22.85 ± 3.85	22.88 ± 4.01	22.81 ± 3.59
Height (m)	1.72 ± 0.10	1.78 ± 0.08 ***	1.63 ± 0.06
Weight (kg)	70.90 ± 14.65	78.23 ± 12.58 ***	59.47 ± 9.38
BMI (kg/m^2^)	23.74 ± 3.63	24.68 ± 3.62 ***	22.28 ± 3.15
Home City (Relative Frequency, %)			
Naples	74.07	44.13	29.94
Avellino	1.29	0.90	0.39
Benevento	0.26	0.13	0.13
Caserta	15.74	10.32	5.42
Salerno	5.55	3.61	1.94
Off-site	3.10	1.81	1.29
Academic Degree Engagement (Percentage, %)			
Bachelor’s Degree in Sport Science	70.84	43.35	27.48
Master’s Degree in Sport Science for Health and Prevention/Sport and Management	28.26	17.03	11.23
Other	0.90	0.51	0.39

Anthropometric characteristics of participants are reported as mean ± standard deviation; home city and academic degree engagement information are reported as percentages (%). Student’s *t* test: *** *p* < 0.001; males vs. females. Abbreviations: BMI, Body Mass Index.

**Table 2:** Physical activity profiles and sports participation characteristics among the study participants.

	Total (N = 775)	Males (N = 472)	Females (N = 303)
Training variables			
Sessions/week	3 (3–4)	4 (3–5) ***	3 (3–4)
Training volume/session (min)	90 (60–120)	120 (90–120) *	90 (60–120)
Training volume/week (min)	360 (240–480)	360 (270–480) ***	270 (180–450)
Experience (y)	4 (2–10)	5 (2–10) **	4 (1–10)
Type of sport	Relative Frequency (%)		
Aquatic	3.10	1.94	1.16
Artistic	8.13	1.68	6.45
Athletic	1.42	0.65	0.77
Ball (team sport)	18.58	16.26	2.19
Combat	1.03	0.90	0.13
Equestrian	0.26	0.00	0.26
Gym	52.13	30.19	21.81
Martial arts	1.81	1.29	0.52
None	10.06	5.03	5.03
Outdoor	1.29	1.03	0.26
Racket	2.19	1.81	0.39

Participants’ sport practice information is reported as median and IQR (interquartile range). IQR is reported as range between 25th and 75th percentile. Type of sport information is reported as percentage (%). Mann–Whitney U test: * *p* < 0.05, ** *p* < 0.01, *** *p* < 0.001; males vs. females.

**Table 3:** Summary of participant responses across the eight validated questionnaires.

			**χ^2^**			
	Males	Females	Value	df	*p*	Cramer’s V
GPAQ	4566.00 (2160.00–7504.00)	4036.00 (2020.00–7048.00)	5.080	2	0.079	0.081
MCS	48.12 (40.02–53.85)	49.00 (40.74–53.80)	1.890	1	0.169	0.049
POMS	48.13 (41.95–55.85)	48.25 (43.25–56.34)	0.952	2	0.621	0.035
PSQI	3.00 (2.00–5.00)	3.00 (2.00–5.00)	0.229	1	0.632	0.017
ODI	0.00 (0.00–3.00)	1.00 (0.00–5.00)	6.060	4	0.195	0.088
MD	10.00 (8.00–11.00)	10.00 (8.00–11.00)	0.925	2	0.630	0.035
IAT	18.5 (9.00–29.25)	18.00 (9.50–29.00)	0.489	2	0.783	0.025
I-DOS	3.00 (2.00–5.00)	3.00 (2.00–5.00)	0.059	1	0.808	0.009

Questionnaire information is reported as median and interquartile range (IQR). IQR is reported as range between 25th and 75th percentile. χ^2^ (*chi-square*) test results for all variables are reported (*p* > 0.05). Abbreviations: Global Physical Activity Questionnaire (GPAQ), Mental Component Summary (MCS), Profile of Mood States 11-items short version (POMS), Pittsburgh Sleep Quality Index (PSQI), Oswestry Disability Index (ODI), Adherence to Mediterranean Diet (MD), Internet Addiction Test (IAT), Italian-Düsseldorf Orthorexia Scale (I-DOS).

**Table 4:** Spearman’s correlation matrix showing the relationships between PA levels, presence of ON symptoms, internet use, and QoL domains.

		GPAQ	MCS	POMS	PSQI	ODI	MD	IAT	I-DOS
GPAQ	Rho *p*-value	_							
MCS	Rho *p*-value	−0.003 0.943	_						
POMS	Rho *p*-value	−0.028 0.431	0.221 *** < 0.001	_					
PSQI	Rho *p*-value	0.019 0.596	0.146 *** <0.001	0.068 0.060	_				
ODI	Rho *p*-value	0.032 0.381	0.009 0.804	0.011 0.763	0.015 0.678	_			
MD	Rho *p*-value	0.055 0.124	0.068 0.057	0.023 0.528	0.055 0.127	0.022 0.533	_		
IAT	Rho *p*-value	0.000 0.999	0.067 0.062	0.170 *** <0.001	0.180 *** <0.001	−0.006 0.861	0.041 0.249	_	
I-DOS	Rho *p*-value	−0.123 *** <0.001	0.019 0.594	0.059 0.098	0.193 *** <0.001	0.042 0.239	−0.049 0.175	0.179 *** <0.001	_

*** *p*-value < 0.001. Abbreviations: Global Physical Activity Questionnaire (GPAQ), Mental Component Summary (MCS), Profile of Mood States 11-items short version (POMS), Pittsburgh Sleep Quality Index (PSQI), Oswestry Disability Index (ODI), Adherence to Mediterranean Diet (MD), Internet Addiction Test (IAT), Italian-Düsseldorf Orthorexia Scale (I-DOS).

## Data Availability

Data generated or analyzed during this study are available from the corresponding author upon reasonable request.

## Supplementary Materials

The following supporting information can be downloaded at: https://www.mdpi.com/article/10.3390/healthcare14030369/s1, File S1: Questionnaire.

## Abbreviations

The following abbreviations are used in this manuscript:

PA	Physical Activity
SB	Sedentary Behavior
MET	Metabolic Equivalent
WHO	World Health Organization
MVPA	Moderate–Vigorous Physical Activity
QoL	Quality of Life
GPAQ	Global Physical Activity Questionnaire
SF-12	Short Form 12 Health Survey
POMS	Profile of Mood States
PSQI	Pittsburgh Sleep Quality Index
ODI	Oswestry Disability Index
MD	Mediterranean Diet
IAT	Internet Addiction Test
I-DOS	Italian-Düsseldorf Orthorexia Scale
GD	General Data
IPAQ	International Physical Activity Questionnaire
HRQoL	Health-Related Quality of Life
PCS	Physical Component Summary
MCS	Mental Component Summary
TMD	Total Mood Disturbance
ON	Orthorexia Nervosa
rho	Spearman’s Correlation
df	Degree of Freedom
IQR	Interquartile Range
