# Supplementary material for: Association Between Physical Activity Level, Quality of Life Determinants, Internet Use, and Orthorexia Among Sport Science Students Living in Naples: An Observational Study
Source: Healthcare (Basel). 2026 Jan 31;14(3):369. doi: 10.3390/healthcare14030369 (PMC12897135; doi:10.3390/healthcare14030369)
Supplement: Supplementary file 1 [file healthcare-14-00369-s001.zip › healthcare-4085562-supplementary/healthcare-4085562-supplementary.pdf]

Questionario per studenti

Questo questionario è anonimo e non raccoglieremo l'indirizzo email perciò le chiediamo di rispondere in modo onesto. Il questionario è suddiviso in più sezioni: ciascuna sezione corrisponde ad una tipologia di questionario. Per favore, completi tutte le sezioni del questionario. Vi ringraziamo in anticipo.

Corso di studi/posizione lavorativa attuale *

- Triennale in Scienze Motorie
- Magistrale in Scienze motorie per la prevenzione ed il benessere
- Magistrale in Scienze e management dello sport e delle attività motorie
- Altro (post laurea)

Provincia di residenza *

Età (in anni) *

Sesso *

- Femmina
- Maschio

Altezza (in metri) *

Peso (in kg) *

Sport praticato attualmente *

Quante volte a settimana ti alleni? *

0 1 2 3 4 5 6 7

Mai — Tutti i giorni

In una seduta di allenamento normale, quante ore ti alleni? *

Svolgi più sedute di allenamento durante la giornata? *

- Sì
- No

A che intensità svolgi l'allenamento? *

- Leggera (1,6<3 MET) attività aerobica che non causa cambiamenti significativi nella frequenza respiratoria.
- Moderata (3<6 MET) attività aerobica che può essere sostenuta mentre si tiene una conversazione.
- Vigorosa (6<9 MET) attività aerobica in cui generalmente non può essere fatta una conversazione.
- Alta (>9 MET) intensità che generalmente non può essere sostenuta per più di 10 minuti.
- Non mi alleno.

Da quanto tempo pratichi questo sport (in anni)? *

0 1 2 3 4 5 6 7 8 9 10

0: Mai praticato uno sport o non mi alleno attualmente — 10: o più di 10 anni

Il tuo lavoro prevede intensa attività fisica durante la quale aumentano notevolmente la respirazione e il battito cardiaco, come trasportare o sollevare carichi pesanti, scavare o eseguire lavori edili per almeno 10 minuti? *

- Sì
- No

Quanti giorni di una normale settimana svolge intensa attività fisica al lavoro? *

0 1 2 3 4 5 6 7

Worst — Best

Quanto tempo trascorre compiendo intensa attività fisica in una normale giornata lavorativa? (in ore) *

0 1 2 3 4 5 6 7 8 9

Worst

10
o più di 10

Il tuo lavoro prevede attività fisica moderata durante la quale aumentano leggermente la respirazione e il battito cardiaco, come un'andatura rapida o trasportare carichi leggeri per almeno 10 minuti? *

- Sì
- No

Quanti giorni di una settimana normale svolge attività fisica moderata a lavoro? *

0 1 2 3 4 5 6 7
Worst Best

Quanto tempo trascorre compiendo attività fisica moderata in una normale giornata lavorativa? (in minuti o in ore) *

Spostamenti: si sposta da un posto all'altro a piedi o in bicicletta per almeno 10 minuti? *

- Sì
- No

Quanti giorni di una settimana normale va a piedi o in bicicletta per almeno dieci minuti per spostarsi da un posto all'altro? (in numero di giorni) *

0 1 2 3 4 5 6 7
Worst Best

Quanto tempo investe in una giornata normale per spostarsi a piedi o in bicicletta da un posto all'altro? (in minuti o in ore) *

Nel tempo libero pratica intensa attività fisica o sport con forte accelerazione della respirazione o del battito cardiaco, come correre o giocare a calcio, per almeno 10 minuti? *

- Sì
- No

Quanti giorni di una settimana normale pratica intensa attività fisica o sport nel tempo libero? (in numero di giorni) *

0 1 2 3 4 5 6 7
Worst Best

Quanto tempo investe in intensa attività fisica o sport in una normale giornata di tempo libero (in minuti o in ore)? *

Nel tempo libero pratica attività fisica moderata o sport con leggera accelerazione della respirazione o del battito cardiaco, come un'andatura sostenuta o andare in bicicletta per almeno 10 minuti? *

- Sì
- No

Quanti giorni di una settimana normale pratica attività fisica moderata o sport nel tempo libero? *

0 1 2 3 4 5 6 7

Worst Best

Quanto tempo investe in attività fisica moderata o sport in una normale giornata di tempo libero? (in minuti o in ore) *

Quanto tempo trascorre seduto o a riposo in una giornata normale? (in minuti o in ore) *

In generale diresti che la tua salute è? *

- Eccellente
- Molto buona
- Buona
- Sufficiente
- Scarsa

Le prossime domande riguardano attività che potresti fare durante una giornata tipica. Attualmente, la tua condizione di salute limita lo svolgimento di queste attività? Se sì, in che misura?

	Sì, molto limitante	Sì, un po' limitante	No, per niente limitante
Attività moderate, come spostare un tavolo, passare l'aspirapolvere o giocare a bowling o golf			
Salire diverse rampre di scale			

Durante le ultime 4 settimane, hai avuto qualcuno dei seguenti problemi con il tuo lavoro o altre attività quotidiane come risultato della tua salute fisica?

	Sì	No
Ottenuto di meno rispetto a quello che ti sarebbe piaciuto ottenere.		
Sei stato/a limitato nel lavoro o in altre attività		

Durante le ultime 4 settimane, hai avuto qualcuno dei seguenti problemi a lavoro o in altre regolari attività quotidiane come risultato di qualche problema emozionale (come sentirti depresso o ansioso)?

	Sì	No
Ottenuto di meno rispetto al desiderato.		
Ho lavorato o svolto attività meno attentamente del solito.		

Durante le ultime 4 settimane, quanto il dolore ha interferito con il tuo lavoro normale (incluso il lavoro fuori casa e in casa)? *

- Per niente
- Un po'
- Moderatamente
- Molto
- Estremamente

Le prossime domande riguardano come ti sei sentito nelle ultime 4 settimane. Per ciascuna domanda, scegli una sola risposta, quella che meglio si addice a come ti sei sentito. Nelle ultime 4 settimane, quanto ti sei sentito...

	Tutto il tempo	La maggior parte del tempo	Una buona parte del tempo	Talvolta	Raramente	Mai
Calmo e appacificato						
Con molta energia						
A terra e triste						

Durante le ultime 4 settimane, quanto spesso la tua salute fisica o problemi emotivi hanno interferito con le tue attività sociali (come far visita ad amici o parenti ecc.)? *

- Tutto il tempo
- Gran parte del tempo
- Talvolta
- Raramente
- Mai

La seguente lista contiene sensazioni che tutti possiamo avere. Leggi con attenzione ciascuna voce e seleziona quello che meglio rappresenta come ti sei sentito nell'ultima settimana.

	Per niente	Un po'	Abbastanza	Molto	Estremamente
Teso	○	○	○	○	○
Arrabbiato	○	○	○	○	○
Stressato	○	○	○	○	○
Infelice	○	○	○	○	○
Pieno di vita	○	○	○	○	○
Con le idee confuse	○	○	○	○	○
Scontento di quello che ho fatto	○	○	○	○	○
Con un tremito diffuso	○	○	○	○	○
Svogliato	○	○	○	○	○
Irritato	○	○	○	○	○

Nell'ultimo mese, di solito a che ora sei andato a dormire la sera (sonno notturno)? *

HH : MM AM

Hour Minutes

Nell'ultimo mese, di solito quanto tempo (in minuti) è stato necessario affinché ti addormentassi di notte? *

Nell'ultimo mese, di solito a che ora di alzavi da letto la mattina? *

HH : MM AM

Hour Minutes

Nell'ultimo mese, quante ore di sonno effettivo hai fatto per notte? (Questo numero può essere diverso rispetto alle ore spese sul letto durante la giornata) *

1 2 3 4 5 6 7 8 9 10

Worst — o più di 10 ore

Nell'ultimo mese, quanto è stato difficoltoso dormire la notte perché non riuscivi ad addormentarti in 30 minuti? *

- No, non nell'ultimo mese
- Almeno 1 volta a settimana
- 1 o 2 volte a settimana
- 3 o più volte a settimana

Quanto stato difficolso dormire la notte perchè non riuscivi ad addormentarti in 30 minuti? *

- No, non nell'ultimo mese
- Almeno 1 volta a settimana
- 1 o 2 volte a settimana
- 3 o più volte a settimana

Quanto stato difficolso dormire la notte perchè ti svegliavi in piena notte o all'alba? *

- No, non nell'ultimo mese
- Almeno 1 volta a settimana
- 1 o 2 volte a settimana
- 3 o più volte a settimana

Quanto è stato difficoltoso dormire la notte perchè avevi bisogno di andare al bagno? *

- No, non nell'ultimo mese
- Almeno 1 volta a settimana
- 1 o 2 volte a settimana
- 3 o più volte a settimana

Quanto è stato difficoltoso dormire la notte perchè non respiravi bene? *

- No, non nell'ultimo mese
- Almeno 1 volta a settimana
- 1 o 2 volte a settimana
- 3 o più volte a settimana

Quanto è stato difficoltoso dormire la notte perchè tossivi o russavi molto forte? *

- No, non nell'ultimo mese
- Almeno 1 volta a settimana
- 1 o 2 volte a settimana
- 3 o più volte a settimana

Quanto è stato difficoltoso dormire la notte perchè sentivi troppo freddo? *

- No, non nell'ultimo mese
- Almeno 1 volta a settimana
- 1 o 2 volte a settimana
- 3 o più volte a settimana

Quanto è stato difficoltoso dormire la notte perchè sentivi troppo caldo? *

- No, non nell'ultimo mese
- Almeno 1 volta a settimana
- 1 o 2 volte a settimana
- 3 o più volte a settimana

Quanto è stato difficoltoso dormire la notte perchè facevi incubi? *

- No, non nell'ultimo mese
- Almeno 1 volta a settimana
- 1 o 2 volte a settimana
- 3 o più volte a settimana

Quanto è stato difficoltoso dormire la notte perchè sentivi dolore? *

- No, non nell'ultimo mese
- Almeno 1 volta a settimana
- 1 o 2 volte a settimana
- 3 o più volte a settimana

Nell'ultimo mese, quanto spesso è stato difficoltoso dormire la notte per altre cause? Se sì, descrivi le cause. Altrimenti scrivi "No" *

Quanto spesso hai preso medicinali per dormire la notte? *

- No, non nell'ultimo mese
- Almeno 1 volta a settimana
- 1 o 2 volte a settimana
- 3 o più volte a settimana

Quanto spesso hai avuto difficoltà a rimanere sveglio mentre guidavi, mangiavi o interagivi con altre persone? *

- No, non nell'ultimo mese
- Almeno 1 volta a settimana
- 1 o 2 volte a settimana
- 3 o più volte a settimana

Quanto è stato difficoltoso per te avere entusiasmo nel fare le cose *

- Per niente difficoltoso
- Un po' difficoltoso
- Abbastanza difficoltoso
- Molto difficoltoso

Come valuti la qualità del tuo sonno in generale? *

- Ottima
- Buona
- Non buona
- Pessima

Quanto è intenso il dolore alla schiena? *

- Lo tollero senza assumere antidolorifici.
- Il dolore è forte ma riesco a gestirlo senza assumere antidolorifici.
- Gli antidolorifici mi danno completo sollievo dal dolore.
- Gli antidolorifici mi danno un moderato sollievo dal dolore.
- Gli antidolorifici mi danno veramente poco sollievo dal dolore.
- Gli antidolorifici non hanno nessun effetto sul dolore, per questo non li assumo.

Quanto il mal di schiena influenza la cura della tua persona (lavarsi, vestirsi ecc.)? *

- Riesco a prendermi cura di me normalmente senza causare maggior dolore.
- Riesco a prendermi cura di me normalmente ma quasto mi causa dolore aggiuntivo.
- Risulta doloroso prendermi cura di me infatti sono lento e molto attento.
- Necessito di aiuto ma riesco a gestire la maggior parte della cura di me.
- Necessito aiuto quotidiano in molti aspetti della cura di me.
- Non mi vesto, mi lavo con difficoltà e rimango a letto.

Quanto il mal di schiena influenza la tua capacità di alzare pesi? *

- Riesco ad alzare persi pesanti senza dolore aggiuntivo.
- Riesco ad alzare pesi pesanti ma questo mi da dolore aggiuntivo.
- Il dolore mi previene dall'alzare pesi pesanti da terra, ma riesco ad alzarli se questi sono posizionati in modo conveniente (es. su un tavolo).
- Il dolore mi previene dall'alzare persi pesanti, ma riesco al alzare pesi più leggeri se sono posizionati in modo conveniente.
- Risco ad alzare solo pesi molto leggeri
- Non sono capace di alzare o portare pesi, per niente.

Quanto il mal di schiena influenza il tuo cammino/camminare? *

- Il dolore non mi previene dal camminare anche lunghe distanze.
- Il dolore mi previene dal camminare più di 1,6 chilometri.
- Il dolore mi previene dal camminare più di 800 metri.
- Il dolore mi previene dal camminare più di 400 metri.
- Riesco a camminare solo utilizzando un bastone o stampelle
- Sto a letto la maggior parte del tempo e devo sforzarmi per raggiungere la toilet.

Quanto tuo dolore influenza la tua capacità di stare seduto/a? *

- Riesco a stare seduto su qualunque sedia per quanto tempo desidero.
- Riesco a stare seduto sulla mia sedia preferita per quanto tempo desidero.
- Il dolore mi previene dallo stare seduto per più di 1 ora.
- Il dolore mi previene dallo stare seduto per più di 30 minuti.
- Il dolore mi previene dallo stare seduto per più di 10 minuti.
- Il dolore mi previene completamente dallo stare seduto.

Quanto il tu dolore influenza la tua capacità di stare in piedi? *

- Riesco a stare in piedi per quanto tempo desidero senza dolore aggiuntivo.
- Riesco a stare in piedi per quanto tempo desidero ma questo mi provoca dolore aggiuntivo.
- Il dolore mi previene dallo stare in piedi per più di 1 ora.
- Il dolore mi previene dallo stare in piedi per più di 30 minuti.
- Il dolore mi previene dallo stare in piedi per più di 10 minuti.
- Il dolore mi impedisce del tutto di stare in piedi.

Quanto il tuo dolore influenza la tua capacità di dormire? *

- Il dolore non mi impedisce di dormire bene.
- Riesco a dormire bene solo assumendo compresse.
- Anche quando assumo compresse dormo per meno di 6 ore a notte.
- Anche quando assumo compresse dormo per meno di 4 ore a notte.
- Anche quando assumo compresse dormo per meno di 2 ore a notte.
- Il dolore mi impedisce del tutto di dormire.

Quanto l tuo mal di schiena infuenza la tua capacità di avere vita sessuale? *

- La mia vita sessuale è normale e non mi causa dolore extra.
- La mia vita sessuale è normale ma questo mi causa dolore extra.
- La mia vita sessuale è abbastanza normale ma è molto dolorosa.
- La mia vita sessuale è severamente compromessa dal dolore alla schiena.
- La mia vita sessuale è quasi assente a causa del dolore alla schiena.
- Il dolore alla schiena mi impedisce totalmente di avere vita sessuale.

Quanto il tuo dolore influenza la tua capacità di avere vita sociale? *

- La mia vita sociale è normale e non mi causa dolore extra.
- La mia vita sociale è normale ma aumenta l'intensità del dolore.
- Il dolore non ha effetto significativo sulla mia vita sociale, se non per la limitazione di interessi che richiedono maggiore energia (es. ballare).
- Il dolore ha limitato fortemente la mia vita sociale e non esco spesso.
- Il dolore spesso limita la mia vita sociale alla mia casa.
- Non ho una vita sociale a causa del dolore alla schiena.

Quanto il tuo dolore influenza la tua capacità di viaggiare?

- Riesco a viaggiare ovunque senza dolore extra.
- Riesco a viaggiare ovunque ma ho dolore aggiuntivo.
- Il dolore è forte ma riesco a viaggiare per più di 2 ore.
- Il dolore restringe i miei viaggi ad una durata inferiore ad 1 ora.
- Il dolore restringe i miei viaggi a quelli brevi e necessari di durata inferiore a 30 minuti.
- Il dolore impedisce qualsiasi viaggio, ad eccezione di quelli verso il medico o l'ospedale.

FRUTTA 1 porzione: 150 g (Esempio: 1 mela, pera o un'arancia; 3 prugne o tre mandarini) *

- < 1 porzione al giorno
- 1-2 porzioni al giorno
- > 2 porzioni al giorno

VERDURA 1 porzione: 100 g (Esempio: 1 piatto di insalata; 2 pomodori; mezza vaschetta di verdura cotta) *

- < 1 porzione al giorno
- 1-2,5 porzioni al giorno
- > 2,5 porzioni al giorno

LEGUMI 1 porzione: 70 g (Esempio: mezza scatoletta di fagioli o ceci o lenticchie o piselli) *

- < 1 porzione a settimana
- 1-2 porzioni a settimana
- 2 porzioni a settimana

CEREALI (pane, pasta, biscotti etc.) 1 porzione: 130 g (Esempi: 1 porzione pasta: 80 g; 4 biscotti frollini: 50 g) *

- < 1 porzione al giorno
- 1-1,5 porzioni al giorno
- > 2 porzioni al giorno

PESCE (eccetto molluschi e crostacei) 1 porzione: 100 g *

- < 1 porzione a settimana
- 1-2,5 porzioni a settimana
- > 2,5 porzioni a settimana

CARNE E SALUMI 1 porzione: 80 g (Esempi: 1 porzione carne: 100 g;1 porzione salumi: 50 g) (esempio: mezza vaschetta di prosciutto) *

- < 1 porzione al giorno
- 1-1,5 porzioni al giorno
- > 1,5 porzioni al giorno

LATTE E LATTICINI 1 porzione: 180 g (Esempi: 1 tazza di latte: 150 g; 1 yogurt: 125 g) *

- < 1 porzione al giorno
- 1-1,5 porzioni al giorno
- >1,5 porzioni al giorno

ALCOL 1 U.A. = 1 bicchiere di vino; 1 lattina di birra *

- < 1 U.A. al giorno
- 1-2 U.A. al giorno
- > 2 U.A. al giorno

OLIO D'OLIVA *

- Occasionalmente
- Frequentemente
- Regolarmente

Domande

	Mai	Raramente	Occasionalmente	Di solito	Spesso	Sempre
Quante volte si è accorto di essere rimasto online più a lungo di quanto intendeva?						
Le capita di trascurare le faccende domestiche per passare più tempo on-line?						
Le capita di preferire l'eccitazione offerta da internet all'intimità con il suo partner?						
Le capita di stabilire nuovi rapporti con altri utenti on-line?						
Accade che le persone attorno a lei si lamentino per la quantità di tempo che passa on-line?						
Accade che i suoi studi risentino negativamente della quantità di tempo che passa on-line?						
Le capita di controllare la sua email prima di fare qualche altra cosa importante?						
La sua resa sul lavoro o la sua produttività sono influenzate negativamente da internet?						
Le capita di stare sulle difensive o di minimizzare quando qualcuno le chiede fa on-line?						
Quante volte si trova a scacciare pensieri negativi sulla sua vita con il pensiero consolatorio di internet?						

Le capita di scoprirsi a pregustare il momento in cui andrà di nuovo on-line?						
Le succede di temere che la vita senza internet sarebbe noiosa, vuota e senza gioia?						
Le capita di scattare, alzare la voce o rispondere male se qualcuno la disturba mentre è collegato?						
Le capita di concentrarsi col pensiero su internet quando non è al computer, o di fantasticare di essere collegato?						
Perde ore di sonno per rimanere fino a tardi collegati davanti al computer?						
Ha già tentato di ridurre la quantità di tempo che passa on-line senza riuscirvi?						
Le capita di scoprirsi a dire "ancora qualche minuto e spengo" quando è on-line?						
Cerca di nascondere quanto tempo passa on-line?						
Le capita di scegliere di passare più tempo on-line anzichè uscire con gli amici?						
Le capita di sentirsi depresso, irritabile, nervoso quando non è collegato, mentre sta benissimo quando è nuovamente davanti al computer?						

Affermazioni

	Sì	No
Per me mangiare cibi sani è più importante del piacere di magiare.		
Ho stabilito delle regole per la mia alimentazione.		
Posso gustare un cibo solo se sono certo che sia sano.		
Cerco di evitare inviti da amici che non siano attenti a una sana alimentazione.		
Trovo giusto, più di altre persone, seguire una dieta sana.		
Se mangio qualcosa di non sano mi sento molto in colpa.		
Ho la sensazione di essere emarginato dai miei amici/colleghi a causa delle miei rigidi criteri dietetici.		
I miei pensieri girano sempre intorno a un’alimentazione sana e regolo, di conseguenza, il corso della mia giornata.		
Mi è difficile infrangere le mie regole dietetiche.		
Quando mangio qualcosa di non sano mi sento depresso.		
