# Supplementary material for: Association Between Physical Activity Level, Quality of Life Determinants, Internet Use, and Orthorexia Among Sport Science Students Living in Naples: An Observational Study
Source: Healthcare (Basel). 2026 Jan 31;14(3):369. doi: 10.3390/healthcare14030369 (PMC12897135; doi:10.3390/healthcare14030369)
Supplement: Supplementary file 1 [file healthcare-14-00369-s001.zip › healthcare-4085562-supplementary/healthcare-4085562-supplementary_EN.pdf]

Machine Translated by Google

Student questionnaire

This survey is anonymous and we will not collect your email address, so we ask you to answer honestly.

The survey is divided into several sections: each section corresponds to a different type of survey.

Please complete all sections. Thank you in advance.

Course of study/current job position *

- Bachelor's Degree in Sports Science
- Master's Degree in Sports Science for Prevention and Well-being
- Master's Degree in Sports and Motor Activities Sciences and Management
- Other (postgraduate)

Province of residence *

Age (in years) *

Gender *

- Female
- Male

Height (in meters) *

Weight (in kg) *

Sport currently practiced *

Machine Translated by Google

How many times a week do you train? *

0 1 2 3 4 5 6 7

May — Everyday

In a normal training session, how many hours do you train? *

Do you do multiple workouts during the day? *

- Yes
- No

At what intensity do you train? *

- Light (1.6<3 MET) aerobic activity that does not cause significant changes in respiratory rate.
- Moderate (3<6 MET) aerobic activity that can be sustained while holding a conversation.
- Vigorous (6<9 MET) aerobic activity in which generally no exercise can be done conversation.
- High (>9 MET) intensity that generally cannot be sustained for more than 10 minutes.
- I don't work out.

How long have you been practicing this sport (in years)? *

0 1 2 3 4 5 6 7 8 9 10

Never practiced one sport or not I currently train — or more than 10 years

Your job involves intense physical activity which significantly increases your breathing and heartbeat, such as carrying or lifting heavy loads, digging or carry out construction work for at least 10 minutes? *

- Yes
- No

How many days in a typical week do you perform intense physical activity at work? *

0 1 2 3 4 5 6 7

Worst — Best

How much time do you spend doing intense physical activity in a normal day? working hours? (in hours) *

0 1 2 3 4 5 6 7 8 9

Worst

Machine Translated by Google

10

or more than 10

Your job involves moderate physical activity during which your blood pressure increases slightly. breathing and heart rate, such as a brisk pace or carrying light loads for at least 10 minutes? *

- Yes
- No

How many days in a typical week do you do moderate physical activity at work? *

0 1 2 3 4 5 6 7

Worst — Best

How much time do you spend doing moderate physical activity in a typical day? working time? (in minutes or hours) *

Travel: Do you walk or cycle from one place to another for at least 10 minutes? *

- Yes
- No

How many days in a normal week do you walk or cycle for at least ten minutes? move from one place to another? (in number of days) *

0 1 2 3 4 5 6 7

Worst — Best

How much time do you spend on a normal day walking or cycling from one place to another? place to the other? (in minutes or hours) *

In his free time he practices intense physical activity or sports with strong acceleration of the breathing or heartbeat, like running or playing soccer, for at least 10 minutes? *

- Yes
- No

How many days in a normal week do you practice intense physical activity or sport over time? free? (in number of days) *

0 1 2 3 4 5 6 7

Worst — Best

Machine Translated by Google

How much time do you spend on intense physical activity or sports in a normal day of free time (in minutes or hours)? *

In your free time, do you practice moderate physical activity or sports with a slight acceleration of breathing or heart rate, such as brisk walking or cycling for at least 10 minutes? *

- Yes
- No

How many days in a normal week do you practice moderate physical activity or sports in your free time? *

0 1 2 3 4 5 6 7

Worst Best

How much time do you spend on moderate physical activity or sports on a typical day of leisure time? (in minutes or hours) *

How much time do you spend sitting or resting on a typical day? (in minutes or hours) *

Overall, would you say your health is? *

- Excellent
- Very good
- Good
- Sufficient
- Poor

The next few questions are about activities you might do during a typical day.
Does your health currently limit your ability to perform these activities? If so, to what extent?

	Yes, very limiting	Yes, a little limiting	No, not limiting at all
Moderate activities, such as moving a table, vacuuming, or playing bowling or golf			
Climb several flights of stairs			

During the last 4 weeks, have you had any of the following problems with your work or other daily activities as a result of your physical health?

Machine Translated by Google

	Yes	No
You got less than you would have liked to get.	◯	◯
You have been limited in your work or other activities	◯	◯

During the last 4 weeks, have you had any of the following problems at work or in other regular daily activities as a result of some emotional problem (such as feeling depressed or anxious)?

	Yes	No
Got less than desired.	◯	◯
I have been working or performing activities less attentively than usual.	◯	◯

During the past 4 weeks, how much has the pain interfered with your normal work (including working outside and at home)? *

- ◯ Not at all
- ◯ A bit'
- ◯ Moderately
- ◯ Very
- ◯ Extremely

The next few questions ask how you've felt over the past four weeks. For each question, choose a single answer, the one that best describes how you've felt. Over the past four weeks, how much have you felt...

	All the time	Most of the time	A good portion of the time	Sometimes	Rarely	May
Calm and peaceful	◯	◯	◯	◯	◯	◯
With a lot of energy	◯	◯	◯	◯	◯	◯
The earth is sad.	◯	◯	◯	◯	◯	◯

During the past 4 weeks, how often did your physical health or emotional problems interfere with your social activities (such as visiting friends or relatives, etc.)? *

- ◯ All the time
- ◯ Most of the time
- ◯ Sometimes
- ◯ Rarely
- ◯ May

The following list contains feelings we can all experience. Read each item carefully and select the one that best represents how you've felt over the past week.

	Not at all	A bit'	Enough	Very	Extremely
Tense	◯	◯	◯	◯	◯
Angry	◯	◯	◯	◯	◯
Stressed	◯	◯	◯	◯	◯
Unhappy	◯	◯	◯	◯	◯
Full of life	◯	◯	◯	◯	◯
Con le idee confuse	◯	◯	◯	◯	◯
Unhappy with what I did	◯	◯	◯	◯	◯
With a widespread tremor	◯	◯	◯	◯	◯
Lazy	◯	◯	◯	◯	◯
Irritated	◯	◯	◯	◯	◯

In the last month, what time did you usually go to sleep at night (nighttime sleep)? *

HH : MM | AM

Hour Minutes

In the last month, how long (in minutes) did it usually take for you to did you fall asleep at night? *

In the last month, what time did you usually get out of bed in the morning? *

HH : MM | AM

Hour Minutes

In the last month, how many hours of actual sleep did you get per night? (This number can be different than the hours spent in bed during the day) *

1 2 3 4 5 6 7 8 9 10

Worst — or more than 10 hours

In the last month, how difficult has it been to sleep at night because you couldn't fall asleep in 30 minutes? *

- ◯ No, not in the last month
- ◯ At least once a week
- ◯ 1 or 2 times a week
- ◯ 3 or more times a week

Machine Translated by Google

How difficult was it to sleep at night because you couldn't fall asleep within 30 minutes? *

- No, not in the last month
- At least once a week
- 1 or 2 times a week
- 3 or more times a week

How difficult was it to sleep at night because you woke up in the middle of the night or at dawn? *

- No, not in the last month
- At least once a week
- 1 or 2 times a week
- 3 or more times a week

How difficult was it to sleep at night because you needed to go to the bathroom? *

- No, not in the last month
- At least once a week
- 1 or 2 times a week
- 3 or more times a week

How difficult was it to sleep at night because you couldn't breathe well? *

- No, not in the last month
- At least once a week
- 1 or 2 times a week
- 3 or more times a week

How difficult was it to sleep at night because you coughed or snored so loudly? *

- No, not in the last month
- At least once a week
- 1 or 2 times a week
- 3 or more times a week

How difficult was it to sleep at night because you felt too cold? *

- No, not in the last month
- At least once a week
- 1 or 2 times a week
- 3 or more times a week

How difficult was it to sleep at night because you felt too hot? *

- No, not in the last month
- At least once a week
- 1 or 2 times a week
- 3 or more times a week

Machine Translated by Google

p

How difficult was it to sleep at night because you had nightmares? *

- No, not in the last month
- At least once a week
- 1 or 2 times a week
- 3 or more times a week

How difficult was it to sleep at night because you felt pain? *

- No, not in the last month
- At least once a week
- 1 or 2 times a week
- 3 or more times a week

In the past month, how often have you had trouble sleeping at night due to other reasons? If yes, please describe the reasons. Otherwise, write "No." *

How often did you take sleeping pills at night? *

- No, not in the last month
- At least once a week
- 1 or 2 times a week
- 3 or more times a week

How often did you have trouble staying awake while driving, eating, or interacting with other people? *

- No, not in the last month
- At least once a week
- 1 or 2 times a week
- 3 or more times a week

How difficult was it for you to have enthusiasm in doing things? *

- Not difficult at all
- A little difficult
- Quite difficult
- Very difficult

C [illegible] l ti l [illegible] dlt lita [illegible] l ? *

Machine Translated by Google

How do you rate the quality of your sleep overall? *

- Excellent
- Good
- Not good
- Terrible

How severe is the back pain? *

- I tolerate it without taking painkillers.
- The pain is severe but I can manage it without taking painkillers.
- Painkillers give me complete pain relief.
- Painkillers give me moderate pain relief.
- Painkillers give me very little pain relief.
- Painkillers have no effect on the pain, that's why I don't take them.

How much does back pain affect your personal care (washing, dressing, etc.)? *

- I am able to take care of myself normally without causing more pain.
- I can take care of myself normally but this causes me additional pain.
- It's painful to take care of myself because I'm slow and very careful.
- I need help but can handle most of my self-care.
- I need daily help with many aspects of self-care.
- I don't get dressed, I wash with difficulty and I stay in bed.

How much does back pain affect your ability to lift weights? *

- I can lift heavy weights without additional pain.
- I can lift heavy weights but it gives me additional pain.
- The pain prevents me from lifting heavy weights off the floor, but I can lift them if they are conveniently positioned (e.g., on a table).
- The pain prevents me from lifting heavy weights, but I can lift lighter weights if they are positioned conveniently.
- I can only lift very light weights
- I can't lift or carry weights at all.

How much does back pain affect your walking/gait? *

- The pain doesn't stop me from walking even long distances.
- The pain prevents me from walking more than a mile.
- The pain prevents me from walking more than 800 meters.
- The pain prevents me from walking more than 400 meters.
- I can only walk using a cane or crutches
- I stay in bed most of the time and have to force myself to reach the toilet.

How much does your pain affect your ability to sit? *

Machine Translated by Google

- I can sit in any chair for as long as I want.
- I can sit in my favorite chair for as long as I want.
- The pain prevents me from sitting for more than 1 hour.
- The pain prevents me from sitting for more than 30 minutes.
- The pain prevents me from sitting for more than 10 minutes.
- The pain completely prevents me from sitting.

How much does your pain affect your ability to stand? *

- I can stand for as long as I want without additional pain.
- I can stand for as long as I want but it causes me additional pain.
- The pain prevents me from standing for more than 1 hour.
- The pain prevents me from standing for more than 30 minutes.
- The pain prevents me from standing for more than 10 minutes.
- The pain prevents me from standing at all.

How much does your pain affect your ability to sleep? *

- The pain doesn't keep me from sleeping well.
- I can only sleep well by taking pills.
- Even when I take pills I sleep less than 6 hours a night.
- Even when I take pills I sleep less than 4 hours a night.
- Even when I take pills I sleep for less than 2 hours a night.
- The pain keeps me from sleeping at all.

How much does your back pain affect your ability to have a sex life? *

- My sex life is normal and doesn't cause me any extra pain.
- My sex life is normal but this causes me extra pain.
- My sex life is pretty normal but it's very painful.
- My sex life is severely affected by back pain.
- My sex life is almost non-existent due to back pain.
- Back pain is completely preventing me from having a sex life.

How much does your pain affect your ability to have a social life? *

- My social life is normal and doesn't cause me any extra pain.
- My social life is normal but the intensity of the pain increases.
- The pain has no significant effect on my social life, other than limiting interests that require more energy (e.g., dancing).
- The pain has severely limited my social life and I don't go out much.
- Pain often limits my social life to my home.
- I don't have a social life because of my back pain.

How much does your pain affect your ability to travel?

Machine Translated by Google

- I can travel anywhere without extra pain.
- I can travel anywhere but I have additional pain.
- The pain is severe but I can travel for more than 2 hours.
- The pain restricts my travel to less than 1 hour.
- The pain restricts my trips to short, necessary ones lasting less than 30 minutes.
- The pain prevents any travel except to the doctor or hospital.

FRUIT 1 serving: 150 g (Example: 1 apple, pear or orange; 3 plums or three mandarins) *

- < 1 serving per day
- 1-2 servings per day
- > 2 servings per day

VEGETABLES 1 portion: 100 g (Example: 1 plate of salad; 2 tomatoes; half a tray of cooked vegetables) *

- < 1 serving per day
- 1-2.5 servings per day
- > 2.5 servings per day

LEGUMES 1 portion: 70 g (Example: half a can of beans or chickpeas or lentils or peas) *

- < 1 serving per week
- 1-2 servings per week
- 2 servings per week

CEREALS (bread, pasta, biscuits, etc.) 1 portion: 130 g (Examples: 1 portion of pasta: 80 g; 4 shortbread biscuits: 50 g) *

- < 1 serving per day
- 1-1.5 servings per day
- > 2 servings per day

FISH (except molluscs and crustaceans) 1 portion: 100 g *

- < 1 serving per week
- 1-2.5 servings per week
- > 2.5 servings per week

MEAT AND COLD CUTS 1 portion: 80 g (Examples: 1 portion of meat: 100 g; 1 portion of cold cuts: 50 g) (example: half a tray of ham) *

- < 1 serving per day
- 1-1.5 servings per day
- > 1.5 servings per day

Machine Translated by Google

MILK AND DAIRY PRODUCTS 1 serving: 180 g (Examples: 1 cup of milk: 150 g; 1 yogurt: 125 g) *

- ◯ < 1 serving per day
- ◯ 1-1.5 servings per day
- ◯ >1.5 servings per day

ALCOHOL 1 AU = 1 glass of wine; 1 can of beer *

- ◯ < 1 AU per day
- ◯ 1-2 AU per day
- ◯ > 2 AU per day

OLIVE OIL *

- ◯ Occasionally
- ◯ Often
- ◯ Regularly

Requests

	May	Rarely Occasionally		Usually	Often Always	
How many times have you found yourself staying online longer than you intended?	◯	◯	◯	◯	◯	◯
Do you find yourself neglecting household chores to spend more time online?	◯	◯	◯	◯	◯	◯
Do you find yourself preferring the excitement of the internet to intimacy with your partner?	◯	◯	◯	◯	◯	◯
Do you find yourself making new connections with other users online?	◯	◯	◯	◯	◯	◯
Do people around you complain about the amount of time you spend online?	◯	◯	◯	◯	◯	◯
Does the amount of time you spend online negatively impact your studies?	◯	◯	◯	◯	◯	◯
Do you ever check your email before doing something else important?	◯	◯	◯	◯	◯	◯
Is your work performance or productivity negatively affected by the Internet?	◯	◯	◯	◯	◯	◯
Do you find yourself getting defensive or downplaying things when someone asks you questions online?	◯	◯	◯	◯	◯	◯
How often do you find yourself chasing away negative thoughts about your life with the comforting thought of the internet?	◯	◯	◯	◯	◯	◯

Machine Translated by Google

Do you ever find yourself anticipating the moment you go online again?	○	○	○	○	○	○
Do you ever fear that life without the internet would be boring, empty, and joyless?	○	○	○	○	○	○
Do you find yourself snapping, raising your voice, or responding rudely if someone disturbs you while you're online?	○	○	○	○	○	○
Do you find yourself thinking about the Internet when you're not at your computer, or fantasizing about being online?	○	○	○	○	○	○
Do you lose hours of sleep by staying late at the computer?	○	○	○	○	○	○
Have you already tried to reduce the amount of time you spend online without success?	○	○	○	○	○	○
Do you ever find yourself saying, "Just a few more minutes and I'll turn it off" when you're online?	○	○	○	○	○	○
Are you trying to hide how much time you spend online?	○	○	○	○	○	○
Do you find yourself choosing to spend more time online rather than going out with friends?	○	○	○	○	○	○
Do you ever feel depressed, irritable, or nervous when you're offline, but feel fine when you're back in front of your computer?	○	○	○	○	○	○

Statements

	Yes	No
For me, eating healthy food is more important than the pleasure of eating.	○	○
I have set some rules for my diet.	○	○
I can only enjoy a food if I am sure it is healthy.	○	○
I try to avoid invitations from friends who aren't careful about healthy eating.	○	○
I think it's right, more than other people, to follow a healthy diet.	○	○
If I eat something unhealthy I feel very guilty.	○	○
I feel like I'm being ostracized by my friends/colleagues because of my strict dietary restrictions.	○	○
My thoughts always revolve around healthy eating, and I plan my day accordingly.	○	○
It's hard for me to break my dietary rules.	○	○
When I eat something unhealthy I feel depressed.	○	○
